# Correlation of HER2, p95HER2 and HER3 Expression and Treatment Outcome of Lapatinib plus Capecitabine in her2-Positive Metastatic Breast Cancer

**DOI:** 10.1371/journal.pone.0039943

**Published:** 2012-07-27

**Authors:** Sae-Won Han, Yongjun Cha, Agnes Paquet, Weidong Huang, Jodi Weidler, Yolanda Lie, Thomas Sherwood, Michael Bates, Mojgan Haddad, In Hae Park, Do-Youn Oh, Keun Seok Lee, Seock-Ah Im, Yung-Jue Bang, Jungsil Ro, Tae-You Kim

**Affiliations:** 1 Department of Internal Medicine, Seoul National University Hospital, Seoul, Korea; 2 Cancer Research Institute, Seoul National University College of Medicine, Seoul, Korea; 3 Monogram Biosciences, Inc., San Francisco, California, United States of America; 4 Center for Breast Cancer, National Cancer Center, Goyang, Korea; 5 Department of Molecular Medicine & Biopharmaceutical Sciences, Graduate School of Convergence Science and Technology, Seoul National University, Seoul, Korea; University of South Alabama, United States of America

## Abstract

**Background:**

Lapatinib plus capecitabine is an effective treatment option for trastuzumab-refractory HER2-positive metastatic breast cancer. We have investigated the correlation between quantitative measures of HER2, p95HER2, and HER3 and treatment outcomes using lapatinib and capecitabine.

**Methods:**

Total HER2 (H2T), p95HER2 (p95), and total HER3 (H3T) expression were quantified in formalin-fixed paraffin-embedded samples using the VeraTag assays. Patients received lapatinib and capecitabine treatment following trastuzumab failure according to the Lapatinib Expanded Access Program. The association between the protein expression levels and clinical outcomes was analyzed.

**Results:**

A total of 52 patients were evaluable. H2T level was significantly higher in responders (median 93.49 in partial response, 47.66 in stable disease, and 17.27 in progressive disease; *p* = 0.020). Longer time-to-progression (TTP) was observed in patients with high H2T [*p* = 0.018, median 5.2 months in high (>14.95) *vs*. 1.8 in low (<14.95)] and high H3T [*p* = 0.017, median 5.0 months in high (>0.605) *vs*. 2.2 in low (<0.605)]. Patients having both high H2T and high H3T had significantly longer TTP [adjusted hazard ratio (HR) 0.38 (95% CI 0.20–0.73), *p* = 0.004] and overall survival [adjusted HR 0.46 (95% CI 0.24–0.89), *p* = 0.020]. No significant association between p95 and response or survival was observed.

**Conclusions:**

These data suggest a correlation between high HER2 and high HER3 expression and treatment outcome, while no significant difference was observed between clinical outcome and p95 expression level in this cohort of HER2-positive, trastuzumab-refractory metastatic breast cancer patients treated with lapatinib and capecitabine.

## Introduction

Human epidermal growth factor receptor 2 (HER2) is overexpressed/amplified in 20%–30% of human breast cancers and plays a key role in breast cancer development and progression [1], [2], [3]. HER2-positive breast cancer is associated with poor clinical outcome, but represents an attractive therapeutic target [4]. Trastuzumab, a humanized monoclonal antibody against the extracellular domain of HER2, in combination with chemotherapy, prolongs survival of early and metastatic HER2-positive breast cancer patients [5], [6], [7]. However, not all patients with HER2-positive metastatic breast cancer show response and many patients eventually develop resistance to trastuzumab within one year [7], [8]. Expression of p95HER2, an NH2-terminally truncated form of HER2 that lacks the extracellular domain for trastuzumab binding, is one of the mechanisms of resistance to trastuzumab [9], [10], [11].

Lapatinib is a small molecule inhibitor of tyrosine kinase activity of HER2 and EGFR. Lapatinib targets the intracellular tyrosine kinase domain and has shown activity against the p95HER2 in addition to the full-length HER2 in preclinical models [9], [10]. Lapatinib in combination with capecitabine significantly improved time to disease progression compared with capecitabine monotherapy in HER2-positive metastatic breast cancer patients that had progressed after trastuzumab therapy [12]. Lapatinib plus capecitabine is currently the standard of care for these patients and is being actively studied in comparison with trastuzumab in various clinical settings. Predictive biomarkers for efficacy and resistance of lapatinib treatment have not been fully understood thus far.

The VeraTag assay technology enables quantitative measurements of ErbB family proteins including HER2, p95HER2, and HER3 in formalin-fixed paraffin-embedded (FFPE) tissue samples. Quantitative measurement of HER2 expression using this assay showed better correlation with TTP and OS compared to central FISH testing in a cohort of HER2 positive metastatic breast cancer patients treated with trastuzumab [13]. In addition, p95HER2, which lacks the trastuzumab binding site, may represent a potential biomarker in identifying patients with trastuzumab resistance [11].

We performed the novel VeraTag assays in a patient cohort of HER2-positive, trastuzumab-refractory metastatic breast cancer treated with lapatinib and capecitabine and examined the correlation between clinical outcome and levels of HER2, p95HER2, and HER3 protein expression.

## Patients and Methods

### Patients and Treatment

The study cohort included patients who agreed to participate in the pharmacogenomic study among the advanced breast cancer patients enrolled in the Lapatinib Expanded Access Program (LEAP) by GlaxoSmithKline at the Seoul National University Hospital (SNUH) and the National Cancer Center Hospital (NCCH) between February 2007 and April 2008. The inclusion criteria were (1) ≥18 years of age with a life expectancy of ≥8 weeks; (2) locally advanced or metastatic breast cancer that had progressed after treatment containing an anthracycline, a taxane, and trastuzumab; (3) HER2 overexpression [3+ on immunohistochemical analysis (IHC) or gene amplification by fluorescence *in situ* hybridization (FISH) as determined by the participating institutions] [14]; (4) Eastern Cooperative Oncology Group (ECOG) performance status (PS) of 0–2 and a cardiac ejection fraction within the institutional range of normal; (5) adequate renal, hepatic, and hematologic function; (6) no contraindication to lapatinib and capecitabine; and (7) at least one measurable lesion. Patients previously treated with lapatinib were excluded.

Treatment consisted of lapatinib at a dose of 1250 mg daily and capecitabine at a dose of 2000 mg/m^2^ in 2 divided doses on day 1 through 14 of a 21-day cycle. Treatment was continued until the patient was dropped from the study due to progression of disease, intolerable toxicity, or the patient’s wish to withdraw. Evaluation of response was performed every 6 weeks according to Response Evaluation Criteria in Solid Tumors (RECIST version 1.0) [15]. Complete or partial responses were confirmed with CT scans taken at least 4 weeks apart. Brain metastasis was evaluated using magnetic resonance imaging. All patients provided written informed consent for LEAP and pharmacogenomic study before study entry. The study protocol was reviewed and approved by the Institutional Review Boards of SNUH and NCCH. Recommendations for the Declaration of Helsinki for biomedical research involving human subjects were also followed.

### Quantitative Analysis of Receptor Expression

Specimens for analysis were derived from archival FFPE tumor samples obtained prior to study entry. One sample per patient was analyzed and most recently acquired tissue was used whenever possible. Anonymized FFPE sections were shipped to Monogram Biosciences (South San Francisco, CA).

The HERmark® HER2 total expression assay is an application of the VeraTag® technology platform designed specifically for breast cancer. VeraTag is a proximity-based method designed to accurately and reproducibly quantify protein expression and protein-protein complexes, including cell surface dimers, in FFPE specimens [16]. The assay has been analytically validated according to the specifications prescribed by the Clinical Laboratory Improvement Amendments (CLIA) and is carried out in a College of American Pathologists-certified clinical reference laboratory at Monogram Biosciences Inc. in South San Francisco, CA [17], [18]. The p95HER2 VeraTag assay was developed on the VeraTag technology platform with a proprietary monoclonal antibody against the carboxy-terminal fragment of HER2. The novel p95HER2 specific antibody and the p95HER2 VeraTag assay have been characterized for sensitivity, specificity, and selectivity over full-length HER2 receptor [11]. The total HER3 expression assay was developed based on the VeraTag technology platform with two HER3 specific monoclonal antibodies. The VeraTag reporter is conjugated to an antibody (anti-HER3 Mouse mAb Ab-6; Labvision, Fremont, CA) that binds a distinct epitope of HER3, while biotin is conjugated to a second antibody (anti-HER3 Mouse mAb clone B9A11; Monogram Biosciences) that recognizes a different HER3 epitope. Streptavidin labeled methylene blue (“molecular scissors”) is coupled via biotin to B9A11. Both antibodies bind epitopes located in the C-terminal region of the HER3 receptor and have no detectable cross-reactivity to other HER receptors. Upon photo-activation, the molecular scissors complex generates a short lived, reactive singlet oxygen species that specifically and precisely cleaves neighboring thio-ether bonds to release fluorescent VeraTag reporter molecules. The liberated VeraTag reporters are collected and quantified by capillary electrophoresis. Quantified signals are normalized to tumor area on the FFPE tissue section. The final value for expression was calculated as [relative fluorescence (RF) concentration] × [capture buffer volume]/[tumor area] giving final units of RF/mm^2^ tumor. Following controls were used in each of the VeraTag assays. For H2T assay, negative: MDA_MB_468, low: MCF7, medium: MDA_MB_453, high: SKBR3, and accuracy: ZR75. For H3T assay, negative: SKOV3, low: MDA_MB_231, medium: MDA_MB_468, high: MDA_MB_453, and accuracy: H1650. For P95 assay, negative: MDA_MB_175, low: MDA_MB_361, medium: clone 1A8 (MCF7 transfected with p95), high: clone 1E2 (MCF7 transfected with p95), and accuracy: UACC 812.

### Statistical Analysis

All VeraTag assays were performed blinded to clinical outcome data, and the treating physicians were blinded to the testing results. After testing, assay results were transmitted to SNUH and NCC before receipt of clinical outcome data at Monogram. Statistical analyses were performed independently at Monogram and SNUH and then compared. Receptor expression levels were compared using the Kruskal-Wallis test and Mann-Whitney *U*-test. Categorical receptor levels (high or low) and response were compared using the Chi-square test. The clinical cut-off values for HER2 and HER3 were determined at the points with the lowest *p-*values in terms of TTP. In the case of p95HER2, however, we could not find any cut-off which was significant in terms of TTP, and the median value was used instead. TTP and OS were calculated using the Kaplan-Meier method. Comparisons of TTP and OS were made with log-rank tests. Statistical analysis of receptor levels and response rate was carried out using Fisher’s exact test. Multivariable analysis of TTP and OS were carried out using the backward stepwise Cox regression model. To adjust for baseline characteristics, age, ECOG PS (0 *vs*. 1–2), hormone receptor status, and previous exposure to capecitabine were included as covariates. Two sided *p*-values of less than 0.05 were considered significant. All statistical analyses were performed using PASW statistics, version 18.0 (SPSS Inc., Chicago, IL).

## Results

### Patient Characteristics and Treatment Outcomes

A total of 52 patients were included in the analysis. Baseline patient characteristics are presented in Table 1. The best overall response of lapatinib plus capecitabine treatment was partial response (PR) in 11 patients (21.2%), stable disease (SD) in 30 (57.7%), and progressive disease (PD) in 11 (21.2%). Clinical benefit rate (PR or SD for more than 6 months) was 38.5%. After a median follow-up duration of 27.8 months (range 11.4–35.0), all of the patients included in the analysis had discontinued treatment: 51 patients due to disease progression and 1 patient due to consent withdrawal. Median time to progression (TTP) was 4.4 months [95% confidence interval (CI) 3.4–5.4] and median overall survival was 14 months (95% CI 8.6–19.4).

**Table 1:** Baseline patient characteristics.

Characteristic	Patients (N = 52)
Median age, years (range)	50 (31–70)
ECOG performance status	
0	8 (15.4%)
1	40 (76.9%)
2	4 (7.7%)
HER2 status	
IHC 3+ or FISH+	52 (100%)
Hormone receptor status	
ER+ and/or PR+	21 (40.4%)
Number of metastatic organs	
1	9 (17.3%)
2	23 (44.2%)
3	10 (19.2%)
≥4	10 (19.2%)
CNS involvement (+)	14 (26.9%)
Median number of prior chemotherapy regimens for metastatic disease (range)	3 (1–9)
Previous exposure to capecitabine	21 (40.4%)

ECOG: Eastern Cooperative Oncology Group, HER2: human epidermal growth factor receptor 2, IHC: immunohistochemistry, FISH: fluorescence *in situ* hybridization, ER: oestrogen receptor, PR: progesterone receptor, CNS: central nerve system.

### HER2, p95HER2, and HER3 Expression

Quantitative expression levels of HER2 (H2T), p95HER2 (p95), and HER3 (H3T) using VeraTag assays were assessable in 52, 51, and 50 patients, respectively. Primary breast tumor tissue was tested in 33 patients (63.5%) and metastatic or relapsed lesion in 19 patients (36.5%). Archival FFPE tissue blocks had been obtained prior to trastuzumab treatment in 39 patients (75%) and after trastuzumab treatment in 13 (25%). Median value of H2T was 48.72 (range 1.37–205.0), p95 1.84 (0–7.96), and H3T 1.01 (0–5.50). H2T level was significantly higher in tissue samples from metastatic or relapsed lesion compared with primary breast tumor (*p* = 0.017 by Mann-Whitney U test; median 65.2 *vs*. 41.6, respectively). H3T level tended to be also higher in metastatic or relapsed lesion (*p* = 0.066; median 1.51 *vs*. 0.96, respectively), whereas p95 level was similar in both groups (*p* = 0.63). However, there was no significant association between the type of tissue used for analysis (primary *vs*. metastatic/relapsed) and TTP or OS. No significant difference was found according to the timing of tissue acquisition (*i.e*., pre- or post-trastuzumab).

### Treatment Outcome and HER2, p95HER2, and HER3 Expression

**Figure 1:**
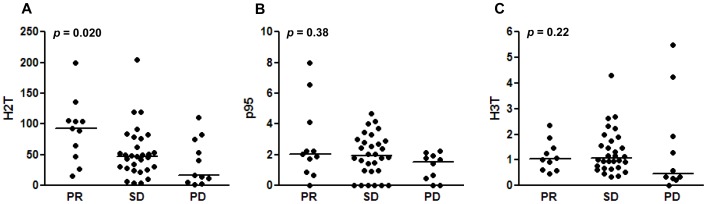
Best overall response and receptor level. *P*-values are by Kruskal-Wallis test. Bars indicate median values.

H2T levels were significantly higher in patients having better response (*p = *0.020 by Kruskal-Wallis test) (Figure 1). Median H2T levels were 93.49 in PR, 47.66 in SD, and 17.27 in PD. P95 and H3T levels were not statistically different according to response.

**Figure 2:**
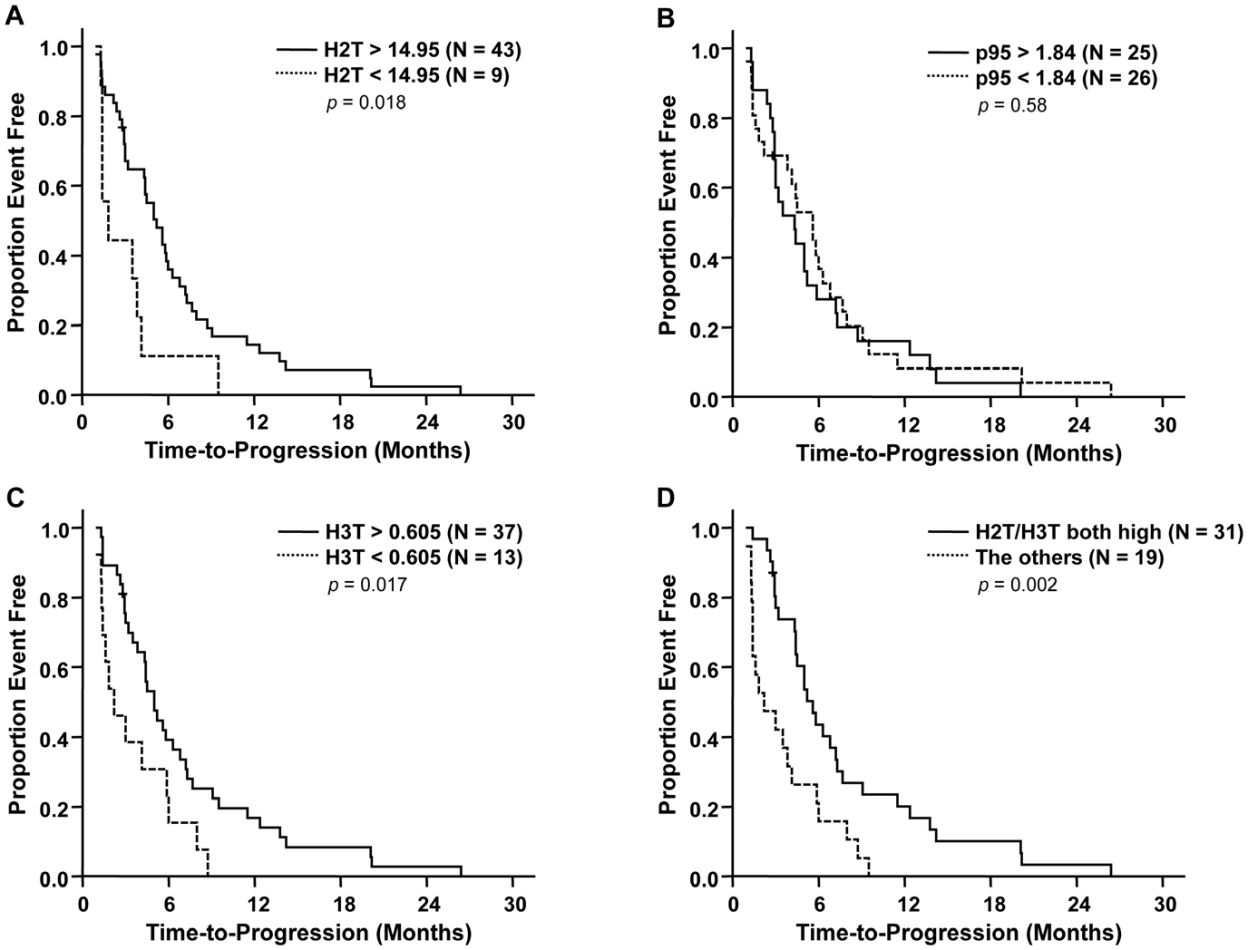
Kaplan-Meier curves of time-to-progression according to HER2 (A), p95HER2 (B), HER3 (C), and HER2/HER3 (D) level. *P*-values by log-rank test.

**Figure 3:**
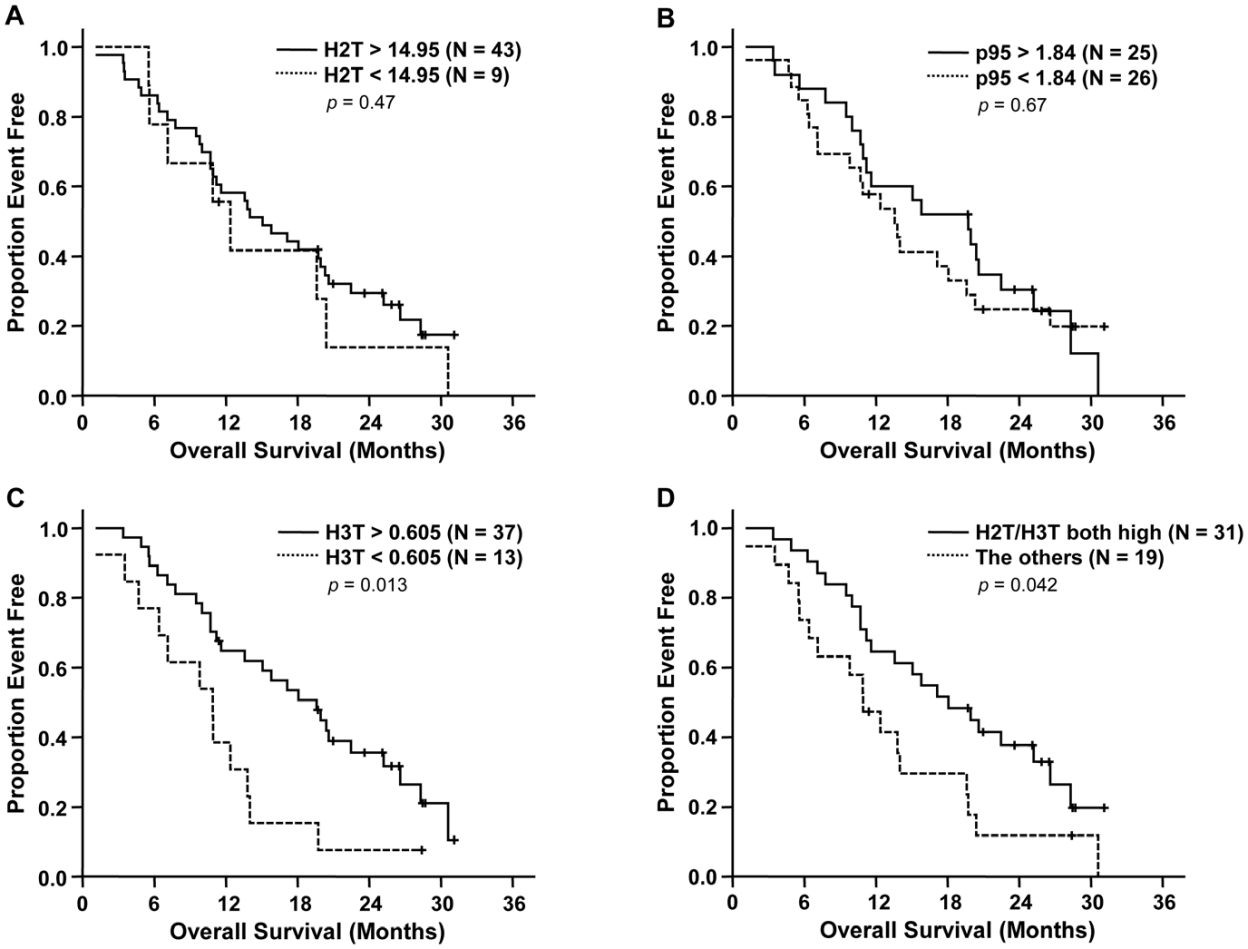
Kaplan-Meier curves of overall survival according to HER2 (A), p95HER2 (B), HER3 (C), and HER2/HER3 (D) level. *P*-values by log-rank test.

Time-to-progression was significantly longer in patients with high H2T [*p* = 0.018, median 5.2 months in high (>14.95) *vs*. 1.8 in low (<14.95)] (Figure 2A). Patients with high H3T also showed longer TTP [*p* = 0.017, median 5.0 months in high (>0.605) *vs*. 2.2 in low (<0.605)] (Figure 2C). In contrast, p95 expression level did not have influence on TTP (*p* = 0.58) (Figure 2B). Similar results were obtained using previously reported cut-offs for H2T and p95 [11], [13]. High H2T (>13.8) had longer TTP [median 5.0 months *vs.* 1.8 in low (<13.8), *p* = 0.047], whereas no difference was observed according to p95 status (*p* = 0.78). In terms of OS, only high H3T was significantly associated with longer OS (*p* = 0.013, median 19.6 months in high *vs.* 10.9 in low) (Figure 3C) and no significant association was found between H2T or p95 levels and OS (Figures 3A and 3B).

To identify a patient subgroup that is most likely to benefit from the treatment, a combination of H2T and H3T levels was evaluated. Patients having both high H2T and high H3T more frequently had PR or SD as best overall response compared with the other patients (Table 2). Notably, only 3.2% of patients having high H2T and H3T had PD as best overall response whereas 47.4% of the other patients did. Patients having both high H2T and H3T had significantly longer TTP (*p* = 0.002; median 5.6 months in high H2T and H3T *vs*. 2.2 in the others) and overall survival (*p* = 0.042; median 18.1 months in high H2T and H3T *vs*. 10.9 in the others) (Figures 2D and 3D). The better TTP and OS in the patients with high H2T and H3T were statistically significant after adjusting for baseline characteristics in multivariate analyses [adjusted hazard ratio (HR) for TTP 0.38 (95% CI 0.20–0.73, *p* = 0.004) and adjusted HR for OS 0.46 (95% CI 0.24–0.89, *p* = 0.020)] (Table 3).

**Table 2:** Treatment response according to receptor level.

		Best overall response [Number of patients (%)]			*p*	Clinical benefit rate (%)	*p*
		PR	SD	PD			
**Overall study population**		11 (21.2)	30 (57.7)	11 (21.2)	-	38.5	-
**HER2**	High (>14.95)	11 (25.6)	26 (60.5)	6 (14.0)	0.013	44.2	0.13
	Low (<14.95)	0 (0.0)	4 (44.4)	5 (55.6)		11.1	
**p95HER2**	High (>1.84)	7 (28.0)	15 (60.0)	3 (12.0)	0.30	36.0	0.65
	Low (<1.84)	4 (15.4)	15 (57.7)	7 (26.9)		42.3	
**HER3**	High (>0.605)	8 (21.6)	25 (67.6)	4 (10.8)	0.023	43.2	0.32
	Low (<0.605)	2 (15.4)	5 (38.5)	6 (46.2)		23.1	
**HER2 and**	Both high	8 (25.8)	22 (71.0)	1 (3.2)	0.001	48.4	0.053
**HER3**	The others	2 (10.5)	8 (42.1)	9 (47.4)		21.1	

**Table 3:** Multivariate analysis of time-to-progression and overall survival.

Time-to-Progression		Adjusted Hazard Ratio(95% CI)	*p*
**Age ≥50**		0.47 (0.24–0.92)	0.028
**Age <50**		1	
**HR positive**		0.51 (0.27–0.97)	0.039
**HR negative**		1	
**HER2 and HER3 both high**		0.38 (0.20–0.73)	0.004
**The others**		1	
**Overall Survival**		**Adjusted Hazard Ratio** **(95% CI)**	***p***
**Prior capecitabine use (+)**		2.2 (1.1–4.3)	0.020
**Prior capecitabine use (−)**		1	
**HER2 and HER3 both high**		0.46 (0.24–0.89)	0.020
**The others**		1	

HR: hormone receptor.

## Discussion

For HER2-positive breast cancer patients, HER2 targeted agents such as trastuzumab or lapatinib are key components of the treatment strategy. However, prior studies have not identified biomarkers that can reliably predict response to lapatinib. In the present study, we have examined the association between quantitative measurements of HER2, p95HER2, and HER3 protein expression and treatment outcome of lapatinib and capecitabine therapy to evaluate their potential roles as predictive biomarkers.

We have found that HER2 levels were higher in patients who showed PR to lapatinib plus capecitabine treatment, and lower in patients having PD as their best overall response. Even though all patients were required to have HER2-positive disease to be eligible for the study treatment, response was different according to quantitative measurement of HER2 expression by the HERmark (VeraTag) assay. This finding is in line with a previous study of HER2-positive metastatic breast cancer patients receiving trastuzumab [13]. In that study, quantitative measurement of HER2 expression using the same HERmark (VeraTag) assay as this study was able to group patients into high and low expression and better treatment results with trastuzumab was observed in the high HER2 expression group [13]. Collectively, the results of the two studies support the importance of quantitative expression level of HER2 in determining the response to HER2-directed treatments, and suggest that the quantitative measurement may improve response prediction in patients receiving such treatments.

Treatment outcome to lapatinib was most favorable in patients with co-expression of high HER2 and HER3 protein in this study. HER3, a member of the EGFR/HER family, is frequently overexpressed in breast cancer with EGFR (HER1) or HER2 overexpression [19]. Although it is kinase inactive, HER3 can be transphosphorylated by EGFR or HER2, and activates the PI3K/AKT survival pathway directly [1], [20]. The formation of ligand-dependent HER2/HER3 heterodimers creates the most mitogenic and transforming receptor complex within the HER3 family [21]. HER3 expression synergistically increases the transforming potency of HER2 [22], and loss of HER3 abolishes the transforming ability of HER2 in HER2 positive breast cancer [23]. Trastuzumab binds to a region of HER2 not involved with receptor dimerization, and cannot block ligand-induced formation of HER2-containing heterodimers or activation of downstream signaling [24], [25]. In addition, high expression of HER3 in tumor tissue was shown to predict early escape from trastuzumab in breast cancer patients [26]. Therefore, HER3 is considered one of the important mechanisms involved in resistance to trastuzumab [20]. In a previous study using VeraTag assay, high HER3 was associated with shorter TTP in patients treated with trastuzumab [27]. In contrast, lapatinib, unlike trastuzumab, can inhibit transphosphorylation of HER3 by HER2 tyrosine kinase and abrogate HER3 signaling activity [10]. The observation that lapatinib was most active in the patient group with high HER2 and HER3 co-expression suggests that it is conceivable lapatinib may overcome trastuzumab resistance through inhibition of HER2/HER3 signaling.

No significant association was observed between p95HER2 expression level and clinical outcome of lapatinib treatment in this study. p95HER2 fragments arise through at least 2 different mechanisms: proteolytic shedding of the extracellular domain of the full-length receptor, and translation of the mRNA encoding HER2 from internal initiation codons [28]. As p95HER2 lacks the epitope recognized by trastuzumab, expression of p95HER2 is hypothesized to engender resistance to trastuzumab. Lack of response to trastuzumab in p95HER2-positive tumors has been shown using the same VeraTag method used in this study [11]. As lapatinib blocks tyrosine kinase activity of the p95HER2 fragments, p95HER2 positive tumors may respond to lapatinib treatment [9], [10], [29]. The lack of association between p95HER2 and treatment outcome in our study is consistent with previous studies and provides additional clinical evidence that lapatinib can inhibit both full-length HER2 and p95HER2. However, it is unclear why patients having higher p95HER2 did not show better treatment outcome considering the positive association observed in the case of higher total HER2 and the outcome in this study. It may be speculated that tumor dependency on HER2 signaling is determined by total HER2 in a larger extent than p95HER2 in this cohort of HER2-positive breast cancer and the tumor dependency on HER2 signaling in turn would determine the treatment result.

The main limitations of this exploratory study include the small sample size, retrospective nature of biomarker testing, and the single-arm, combination treatment with capecitabine. Despite the limitations, we have identified a patient subgroup with high HER2 and HER3 expression that showed better treatment outcome to lapatinib and capecitabine, and showed that the treatment was active regardless of p95HER2 level. We believe these findings merit further investigation in future studies including validation of the cut-off values used herein.

In conclusion, the present study suggests a correlation between the co-expression of high HER2 and high HER3 levels and improved treatment outcomes in patients with trastuzumab-refractory MBC who were treated with lapatinib plus capecitabine. The lack of association between p95HER2 and treatment outcome is consistent with the hypothesis that lapatinib can inhibit both full-length HER2 and p95HER2.
